# Macrothrombocytopenia, renal dysfunction and nephrotic syndrome in a young male patient: a case report of MYH9-related disease

**DOI:** 10.1590/2175-8239-JBN-3879

**Published:** 2018-05-17

**Authors:** Gabriela Sevignani, Giovana Memari Pavanelli, Sibele Sauzem Milano, Bianca Ramos Ferronato, Maria Aparecida Pachaly, Hae II Cheong, Mauricio de Carvalho, Fellype Carvalho Barreto

**Affiliations:** 1 Universidade Federal do Paraná, Departamento de Medicina Interna, Divisão de Nefrologia, Curitiba, PR, Brasil.; 2 Universidade Federal do Paraná, Hospital das Clínicas, Departamento de Medicina Interna, Curitiba, PR, Brasil.; 3 Seoul National University, Children's Hospital, Department of Pediatrics, Seoul, Korea.

**Keywords:** Renal Insufficiency, Chronic, Thrombocytopenia, Nephrotic Syndrome, Genetic Diseases, Inborn, Rare Diseases, Insuficiência Renal Crônica, Trombocitopenia, Síndrome Nefrótica, Doenças Genéticas Inatas, Doenças Raras

## Abstract

*MYH9*-related disease is an autosomal dominant disorder caused by mutations of the *MYH9* gene, which encodes the non-muscle myosin heavy chain IIA on chromosome 22q12. It is characterized by congenital macrothrombocytopenia, bleeding tendency, hearing loss, and cataracts. Nephropathy occurs in approximately 30% of MYH9-related disease in a male patient carrier of a *de novo* missense mutation in exon 1 of the *MYH9* gene [c.287C > T; p.Ser(TCG)96(TTG)Leu]. He presented all phenotypic manifestations of the disease, but cataracts. Renal alterations were microhematuria, nephrotic-range proteinuria (up to 7.5 g/24h), and rapid loss of renal function. The decline per year of the glomerular filtration rate was 20 mL/min/1.73m^2^ for five years. Blockade of the renin-angiotensin system, the only recommended therapy for slowing the progression of this nephropathy, was prescribed. Although MYH9-related disease is a rare cause of glomerulopathy and end-stage renal disease, awareness of rare genetic kidney disorders is essential to ensure accurate diagnosis and proper management of orphan disease patients.

## RESUMO

A doença relacionada ao *MYH9* é um distúrbio autossômico dominante causado por mutações no gene *MYH9* que codifica a cadeia pesada da miosina não muscular IIA no cromossomo 22q12. Ela é caracterizada por macrotrombocitopenia congênita, tendência a sangramento, perda auditiva e catarata. A nefropatia ocorre em aproximadamente 30% dos pacientes. O presente artigo relata o caso de um paciente com doença relacionada ao MYH9 portador de mutação missense *de novo* no exon 1 do gene *MYH9* [c.287C > T; p.Ser(TCG)96(TTG)Leu]. Com a exceção de catarata, o paciente apresentou todas as manifestações fenotípicas da doença. As alterações renais incluíram micro-hematúria, proteinúria nefrótica (até 7,5 g/24h) e perda rápida da função renal. O declínio anual da taxa de filtração glomerular foi de 20 mL/min/1,73 m^2^ durante cinco anos. Foi receitado bloqueio do sistema renina-angiotensina, a única terapia recomendada para retardar a progressão dessa nefropatia. Embora a doença relacionada ao MYH9 seja uma causa rara de glomerulopatia e doença renal terminal, a conscientização sobre distúrbios genéticos renais raros é essencial para garantir o diagnóstico preciso e o manejo adequado dos pacientes com tal doença órfã.

## INTRODUCTION

*MYH9*-related disease (*MYH9*-RD) is a genetic disorder of autosomal dominant inheritance caused by mutations of the *MYH9* gene, which encodes the non-muscle myosin heavy chain IIA (NMMHC-IIA) on chromosome 22q12. Around 200 affected families have been described in the literature, which suggest a very low prevalence of this disease.

*MYH9*-RD is characterized by congenital macrothrombocytopenia, leading to bleeding tendency, along with cytoplasmic inclusion bodies within leukocytes (Döhle-like inclusions), sensorineural deafness, cataracts, and nephropathy. The latter usually presents at a juvenile age with proteinuria, sometimes causing nephrotic syndrome, with or without microhematuria. It often progresses to end-stage renal disease (ESRD).^1^

Herein, we sought to describe the case of a young male patient affected by *MYH9*-RD that developed nephrotic-range proteinuria, microhematuria, and rapid loss of kidney function.

### CASE DESCRIPTION

**Figure 1:**
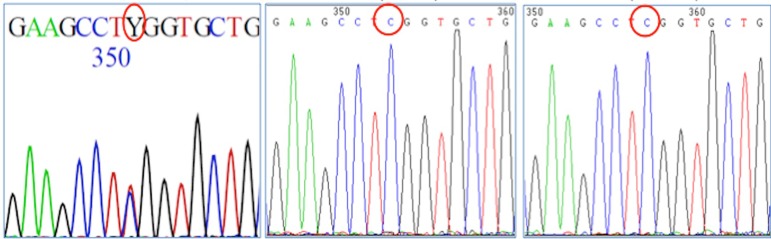
Molecular test of the index case and of his parents; Footnote: a *de novo* heterozygous c.287C>T in exon 1 of *MHY9* [p.Ser(TCG)96(TTG) Leu] was detected (i). His father (ii) and his mother (iii) did not have the mutation.

A twenty-year-old male has been followed up at the Clinic Hospital of Federal University of Paraná due to medical history of epistaxis, ecchymosis, and petechiae since infancy. At first, Bernard-Soulier syndrome was suspected due to macrothrombocytopenia and tendency of bleeding. When he was 17 years old, hearing loss and hypertension were detected along with mild renal failure, microhematuria and nephrotic-range proteinuria. Renal biopsy could not be performed due to risk of bleeding (platelets count: 7000/µL). Cataracts were excluded by ophthalmological evaluation. Due to the clinical suspicion of *MYH9*-RD, genotyping of the patient and of his parents was performed. A *de novo* missense mutation in exon 1 of the *MYH9* gene [c.287C > T; p.Ser(TCG)96(TTG)Leu] was detected (Figure 1). Actually, neither his parents nor his brother and sister had clinical manifestation of *MYH9*-RD. Enalapril (20 mg/day) was initiated for renal protection. The patient did not adhere to treatment and was lost to follow-up. Two years later, he returned to the outpatient clinic complaining of foamy urine, peripheral edema, and hypertension (160/120 mmHg). Laboratory tests detected worsening of renal function and persistent proteinuria. Table 1 shows the evolution of laboratory parameters during the follow-up.

**Table 1:** Laboratory evolution.

Laboratory	June 2012	March 2013	June 2015	June 2017
creatinine (mg/dL)	0.9	1.2	1.7	3.4
eGFR (mL/min/1.73m^2^)	126	88.4	57.2	24.4
urea (mg/dL)	34	27	32	80
proteinuria (g/24h)	NA	7.5	5.5	5.7
albumin (g/dL)	3.4	3.1	3.5	3.1
cholesterol (mg/dL)	185	205	249	241
platelets count (n/µL)	4000	7000	6500	3000

Abbreviations: eGFR: CKD-EPI estimated glomerular filtration rate; NA: not available.

## DISCUSSION

*MYH9*-RD is characterized by congenital macrothrombocytopenia associated with variable degrees of sensorineural hearing loss, pre-senile cataract, and renal disease. Nephropathy occurs in approximately 30% of the patients with *MYH9*-RD and has a progressive and severe evolution. It usually presents at a juvenile age with proteinuria, sometimes causing nephrotic syndrome, with or without microhematuria. Our patient presented all clinical manifestation of *MYH9*-RD, but cataract. In most patients, nephropathy progresses to ESRD before the fourth decade of life.^1^

A genotype-phenotype correlation has been recognized in *MYH9*-RD. A higher incidence and a worse prognosis of kidney impairment have been associated with mutations affecting the head domain of NMMHC-IIA, compared with mutations in tail domain.^2^ Most patients with *MYH9*-RD present an autosomal dominant inheritance, and around 30% of them have a *de novo* mutation.^1^ Our patient presented a de novo missense mutation in exon 1 of *MYH9* [c.287C > T; p.Ser(TCG)96(TTG)Leu] in the head domain. To date, more than 30 mutations within the 40 exons of the *MYH9* gene have been detected, among them the one of our patient.^3^ In agreement with the genotype-phenotype correlation, our patient developed a rapid deterioration of renal function. The decline per year of the glomerular filtration rate was 20 mL/min/1.73m^2^ during the last five years.

Due to the overlap of clinical manifestations, *MYH9*-RD associated with renal impairment was considered a variant of Alport syndrome, designated as Fechtner syndrome. Recently, these syndromes were recognized as distinct disorders. They can be distinguished by the presence of thrombocytopenia, the hallmark of *MYH9*-RD and not a feature of Alport syndrome. Moreover, the latter is caused by mutations in the *COL4A3, COL4A4*, and *COL4A5* genes, leading mainly to alterations in the glomerular basement membrane.^4^ The clinical features together with the presence of a pathogenic mutation in the *MYH9* gene allowed a prompt and reliable diagnosis of *MYH9*-RD.

Renal biopsy is not usually performed in *MYH9*-nephropathy because of the risk of bleeding, reserved for cases in which the differential diagnosis is necessary. Renal histopathological findings are variable and unspecific, encompassing mesangial expansion or proliferation and segmental glomerulosclerosis. Electron microscopy commonly reveals glomerular basement membrane thickening and podocyte foot process effacement^1^. The pathogenesis of *MYH9*-nephropathy is not completely understood. NMMHC-IIA is an important component of podocyte foot process. Thus, *MYH9*-nephropathy may result from an alteration in the podocyte cytoskeleton.^5^

Blockade of the renin-angiotensin system might be effective in reducing proteinuria and slowing the progression of *MYH9* nephropathy.^6^ As our patient did not adhere to the treatment, we could not evaluate the efficacy of this strategy, though.

To the best of our knowledge, this is the first case of *MYH9*-nephropathy described in Brazil. The learning points of this case need to be highlighted. In case of macrothrombocytopenia of uncertain diagnosis, urinalysis must be performed and proteinuria should be monitored to start renin-angiotensin system blockage as early as possible. Genotyping is a valuable tool for guiding diagnosis and prognosis. Finally, awareness of rare genetic kidney disorders is essential to ensure accurate diagnosis and proper management of orphan disease patients.
